# Methane-generating ammonia oxidizing nitrifiers within bio-filters in aquaculture tanks

**DOI:** 10.1186/s13568-018-0668-2

**Published:** 2018-08-28

**Authors:** Barry Kamira, Lei Lei Shi, Li Min Fan, Cong Zhang, Yao Zheng, Chao Song, Shun Long Meng, Geng Dong Hu, Xu Wen Bing, Zhang Jia Chen, Pao Xu

**Affiliations:** 1 Nanjing Agricultural University, 1 Weigang, Nanjing, 210095 Jiangsu People’s Republic of China; 2 Wuxi Fisheries College, 9 East Shan Shui Road, Binhu District, Wuxi, 214081 Jiangsu People’s Republic of China; 3 Key Laboratory of Freshwater Fisheries Eco-Environment Monitoring Center of Lower Reaches of Yantze River, Ministry of Agriculture; Fishery Environmental Protection Department, Freshwater Fisheries Research Center (FFRC), Chinese Academy of Fishery Sciences (CAFS), Wuxi, Jiangsu People’s Republic of China; 4 Present Address: Freshwater Fisheries Resources Center (FFRC), Chinese Academy of Fisheries Sciences (CAFs), Wuxi, People’s Republic of China

**Keywords:** Bio-filters, AOB, DGGE, 16S rRNA, Methyl ammonia oxidizing nitrifiers, Environmental characteristics

## Abstract

The discovery of aerobic and anammox bacteria capable of generating methane in bio-filters in freshwater aquaculture systems is generating interest in studies to understand the activity, diversity, distribution and roles of these environmental bacteria. In this study, we used microbial enrichment of bio-filters to assess their effect on water quality. Profiles of ammonia-oxidizing bacterial communities generated using nested PCR methods and DGGE were used to assess the expression of 16S rRNA genes using DNA sequencing. Five dominant ammonia-oxidizing bacterial strains–clones; KB.13, KB.15, KB.16, KB.17 and KB.18—were isolated and identified by phylogenetic analysis as environmental samples closely related to genera *Methylobacillus, Stanieria, Nitrosomonas,* and *Heliorestis*. The methyl ammonia-oxidizing microbes thereby found suggest a biochemical pathway involving electron donors and carbon sources, and all strains were functional in freshwater aquaculture systems. Environmental parameters including TN (2.69–20.43); COD (9.34–31.47); NH_4_^+^-N (0.44–11.78); NO_2_^−^N (0.00–3.67); NO_3_^−^N (0.05–1.82), mg/L and DO (1.47–10.31 µg/L) assessed varied in the ranges in the different tanks. Principal component analysis revealed that these water quality parameters significantly influenced the ammonia oxidizing microbial community composition. Temperature rises to about 40 °C significantly affected environmental characteristics—especially DO, TN and NH_4_^+^-N—and directly or indirectly affected the microbial communities. Although the nested PCR design was preferred due to its high sensitivity for amplifying specific DNA regions, a more concise method is recommended, as an equimolar mixture of degenerate PCR primer pairs, CTO189f-GC and CTO654r, never amplified only 16S rRNA of ammonia-oxidizing bacteria.

**Electronic supplementary material:**

The online version of this article (10.1186/s13568-018-0668-2) contains supplementary material, which is available to authorized users.

## Introduction

Maintenance of optimal water quality and removal of nitrogen compounds poses challenges to aquaculture (van Kessel et al. 2010). Bio-filtration is an important separation process employed to convert toxic nitrogen metabolites into less toxic forms (Crab et al. 2007), although the identity of the micro-organisms responsible for this conversion has not been well characterized (Tal et al. 2003; van Kessel et al. 2010). In bio-filtration systems, the pollutants are removed by biological degradation rather than physical filtration (Rijn 1996; Hargreaves 1998). And diverse microbial community structures can facilitate decomposition of chemical pollutants and improve water quality (Ibekwe et al. 2007).

Pioneering studies of the ammonia oxidizing bacteria (AOB) have suggested that these nitrifiers fall within the beta- and gamma-*Proteobacteria* sub-divisions. Thus, most molecular studies are limited to and focus on understanding of the two phylogenetic groups (Egli et al. 2001; Konneke et al. 2005). Denitrification through facultative anaerobic bacteria utilizing organic (heterotrophic) or inorganic (autotrophic) compounds as electron sources to reduce nitrate to nitrogen gas, creates further challenges such as nitrous oxide release (Hui et al. 2014). Coupled with the requirement of an external electron source, this has prevented the full-scale commercial application of the process. Anaerobic ammonia oxidation (anammox) is another pathway that allows oxidation of ammonia into nitrite under anoxic conditions, yielding molecular nitrogen (Kowalchuk et al. 1997). Although anammox has been considered economically viable, with limited oxygen requirement for the process (Jetten et al. 2001) and responsible for 30–50% nitrogen loss (Brandes et al. 2007; Lam et al. 2009), such findings were obtained mainly in the marine sector (Thamdrup and Dalsgaard 2002; Kuypers et al. 2003; Schmid et al. 2007). However, our interests lie in freshwater aquaculture systems. As suggested by Lahav et al. (2009), presence of anammox in waste treatment plants revealed that these microbes could be present in freshwater aquaculture systems. Thus, the discovery of anammox bacteria in biological filters in freshwater aquaculture generates interest in understanding the activity, diversity, and distribution of these microbes in the environment (Ward 2015). In addition, methyl ammonia oxidation is a newly discovered pathway which generates methane as a byproduct within the microbial nitrogen cycle. This pathway allows ammonia to be oxidized to nitrite or nitrate under anoxic conditions (Zhu and Chen 1999; Saucier et al. 2000) perturbing carbon sequestration.

Methane is known to play an important role in the carbon cycle of freshwater and soil environments (Hanson 1980); dimictic lakes (Rudd and Hamilton 1975), temperate wetlands (Harriss et al. 1982; Bartlett et al. 1985), and coastal sediments (Sansone and Martens 1981) that undergo partial or complete anoxia. In larger body water like lakes and oceans, the carbon cycle is fundamentally essential with similar differences, such as they do not undergo anoxia since re-mineralization proceeds aerobically without producing methane, except in shallow sediment or enclosed basin environments (Ward 1987). However, in shallow enclosed aquatic production systems like ponds, and tanks, methane gas is highly observed in the environments owing to the fact chemical oxygen demand (COD) and biological oxygen demand (BOD) within the intensive production cycles trigger production of methane (Fan et al. 2018). In Life Cycle Assessment data: (LCA), several studies have been performed on the green house gas (GHG)—emission of aquaculture systems, although most data majorly covers energy use or global warming potential of large farms. The impact on global warming on the production of specific products has been assessed through quantification of emissions of: carbon dioxide (CO_2_), methane (CH_4_), and nitrous oxide (N_2_O) (Mascha et al. 2013).

For aquaculture production chains fish feed is typically the most dominant factor in GHG-emissions. The emissions are mainly determined by the amount of feed needed for the production of a kg of fish (Feed Conversion Ratio: FCR), and FCR for tilapia, Atlantic salmon and Rainbow trout are around 1.7, 1.1 and 1.5 respectively. For the latter two, production feed accounts for, on average, 87% of total GHG emissions (Pelletier and Tyedmers 2007, 2010).

In an anaerobic environment, carbon is converted into methane (Heng et al. 2017). In aquaculture, methane formation occurs mainly in mud layers in intensive ponds, e.g. the anaerobic mud layer in pangasius cultivation (Mascha et al. 2013). Methane emissions, have not been widely covered, and an estimation made in the Mascha et al. study suggested around 5% of the fish feed could be converted into manure. 50% of the manure content existed as carbon that was converted in totality of 3.3% CH_4_ in an anaerobe environment. In their study, they observed that the use of fish feed in the pangasius production estimated at 2.1 million tons, resulted in 70 million kg of CH_4_ production.

Furthermore, Burg van den et al. 2012, also revealed that nitrous oxide is released during microbial transformation of nitrogen in the soil or in manure (i.e. nitrification of NH_3_ into NO_3_^−^ and incomplete denitrification of NO_3_^−^ into N_2_) are engulfed in nitrate fertilizer production for feed ingredients.

Although methanogenic bacteria have been isolated from marine sediment (Sower and Ferry 1983), oceanic environment (Ward 1987), freshwater sediment and sewer outfalls (Whittenbury et al. 1970) in the past, their occurrence is scarcely recorded in freshwater tanks and pond aquaculture systems. Considering evidence of the distribution of methane and nitrification activities in intensive production systems, and related similarities between nitrifiers and methanotrophs, we hypothesized that some nitrifiers in tank and intensive pond production systems are involved in methane cycles. Furthermore, the role of conventional methanotrophs may be partially fulfilled by other kinds of oxidizing bacteria, thereby justifying studies to understand ammonia oxidizing (AO) nitrifier types involved in methane metabolism.

In this study, we aimed at using filters with attached biomass on the filter-media (bio-filter) that have granular activated carbon (GAC) to understand the dynamics of freshwater fish aquaculture water treatment together with microbes (pro-biotic) in tank production from July to October 2017 at the Freshwater Fisheries Research Center (FFRC), Wuxi, China. The objectives were to characterize and identify the methane-generating AO nitrifier communities that allow ammonia oxidation under (1) aerobic and (2) anoxic conditions, and to correlate the effects of bio-filter technologies on the activity, distribution, presence and succession of the microbial communities in tank production systems. Our results will promote design of efficient methods for nitrogenous compound removal in aquaculture.

## Materials and methods

### Tank facilities and experimental designs

The experiment was conducted at the Wuxi Fisheries College of Nanjing Agricultural University from July to October 2017, in outdoor glass fiber cylindrical tanks; [diameter = 135 cm, height = 95 cm], under a sunray overhead mesh. Twelve tanks were used in this experiment. Each tank contained water volumes of approximately 1000 L de-chlorinated freshwater. Three tanks served as control (Ctrl), with no bio-filters and contained 10 experimental fish. The remaining 9 tanks had a string of 7 submerged bio-filters suspended in the tanks and contained 10 experimental fish. Of the 9 tanks, 3 contained bio-filters, and bacteria from the wild; this was labeled as the environmental microbial tank (*EsB*); another set of 3 tanks contained bio-filters, environmental microbes and were enriched with *Pseudomonas* bacteria strain (*PsB*); and the final 3 tanks contained environmental microbes and were enriched with Lactic acid bacterial strain (*LsB*). All tanks were fitted with air-stones connected to an air pump which were operated only when oxygen levels reduced to below 3–4 mg/L. At 3 instances de-chlorinated freshwater was added to each tank to maintain the water levels. All of the four treatments were done in triplicate.

### Fish stocking, bacterial enhancements and tank management

Healthy mixed-sex Nile tilapia (*Oreochromis niloticus*) fingerlings were obtained from the Freshwater Fisheries Research institute’s breeding center in Yi Xing, Wuxi, China in June 2017. Before the trials, fingerlings were acclimatized in cylindrical white plastic tanks (1000 L) for 2 weeks, and healthy fish with body weight of 8 ± 0.3 g were selected and randomly stocked into 12 tanks. Commercial pelleted feed (Ningbo Tech Bank Co. Ltd) containing 30% crude protein, 15% fibre, 18% ash content and 12% moisture content was used to feed fish twice a day (at 0800 and 1500 h) to achieve apparent satiation for the entire study period. At the start of each week, feed rations were revised after calculations of food conversion ratio (FCR) with approximately similar quantities of feed being applied to each tank. Feed inputs were recorded weekly for each tank.

Microbial strains, i.e. the *Pseudomonas* and *lactic acid bacteria* strains (obtained from FFRC, environmental laboratory), with the former being isolated from the surface sediment of a tilapia pond in the southern district of Freshwater fisheries research center and identified by the 16S rDNA homology analysis (unpublished report) and the latter purchased from Jiangsu Green Biotechnology Co., Ltd (Yangzhou, China) were separated, enriched and cultured in the institute’s Eco-environment laboratory. Heterotrophic nitrification media of two batch reactors (with working volumes of 100 mL) were used to culture the mixture under agitation (150 rpm at 30 °C) as described by Zhang et al. (2011). Nutrient media contained: (NH_4_)_2_SO_4_—(0.24 g/L); K_2_HPO_4_·3H_2_O—(6.50 g/L); MgSO_4_·7H_2_O (2.50 g/L); NaCl—2.50 g/L; MnSO_4_·H_2_O—(0.04 g/L); FeSO_4_·H_2_O—(0.05 g/L); and C_4_H_4_Na_2_O_4_—(2.17 g/L). The changes in the effluent water quality—including nitrite nitrogen (NO_2_^−^-N), nitrate nitrogen (NO_3_^−^-N) and chemical oxygen demand (COD)—were measured after 3 days to ensure the absolute removal of nitrite and ammonia. Ten milliliter of the above liquid containing microbes from each culture were then placed in the experimental tanks for further studies. The tank enhancement with the bacterial microbes was done every 14 days starting in first week of July 2017; samplings were done in August, September and October 2017.

### Water quality assessments

To assess need for operating the aerators, pH, DO, temperature, and light intensity were determined thrice a week nearest to the submerged bio-filters after 1800 h using a portable pH meter (Sanxin PHB-1) and a Lei Ci multi-parameter water quality instrument (JPBJ-608 INESA). Water samples were collected weekly; NH_4_^+^-N, TN, COD, NO_2_^−^-N and NO_3_^−^-N concentrations were measured according to Standard Methods. Water was sampled 6 times per month, while the bacterial strains were enhanced twice every 14 days and sampled once. Using standard analytical methods (APHA 1988), Nessler’s reagent spectrophotometry; Titrimetric; N-(1-naphthalene)-di-amino ethane spectrophotometry, and ultraviolet spectrophotometry methods were engaged to measure NH_4_^+^-N, TN, COD, NO_2_^−^-N and NO_3_^−^-N environmental parameters respectively.

### Microbial sampling procedure and DNA extraction

Aquatic microbial samples were collected at points nearest to the fixed bio-filters and 5 cm below the surface using a Vandorn water sampler (1 L). Samples of 300 mL of water from each tank were siphoned into a sterilized glass bottle and immediately taken to the laboratory for filtration through GF/C filter papers under vacuum following the manufacturer’s procedures (Mo Bio Power Water^®^ DNA Isolation Kit). This was done in triplicate; the filters were stored in a freezer at − 80 °C for further analyses. Further, from each tank, the fourth bio-filter was raised and 3–5 strings were removed from the filter for analysis of the bacterial community attached to the filter.

Genomic DNA was extracted following the Mo Bio Lab Inc. methods using the protocol involving 24 steps with initial bead beating homogenizing lysis step, patented inhibitor removal, generation of cDNA, and purification using the Power Clean DNA Clean up Kit. Each sample was extracted in triplicate and extracts from the same samples pooled together. The extracted DNA was stored at − 20 °C until use.

### PCR amplifications and pyrosequencing

Nested-PCR for 16S rRNA gene amplification was used with modification of the method of Ziembinska et al. 2009. In brief, the first round of PCR, enabling a partial amplification of the 16S rRNA gene belonging to the ammonia-oxidizing β-*Proteobacteria*, was performed using bacterial primers CTO189F-ABC-GC, [5′-CCG CCG CGC GGC GGG CGG GGC GGG GGC ACG GGG GGA GRA AAG YAG GGG ATCG-3′] and CTO654r, [5′-CTAGCYTTGTAGTTTCAAACGC-3′] (Kowalchuk et al. 1997; Ziembinska et al. 2009; Zhao et al. 2016). PCR was carried out in an Applied Bio-System (Life Technologies) Veriti^®^ 96-well thermal cycler with 50-µL reaction volumes. The reaction mixture contained 10 × AmpliTaq Gold PCR buffer (5 µL); MgCl_2_ (4 µL); Enhancer (2 µL); 2 mM dNTP mix (4 µL); forward and reverse primers (each 1 µL); AmpliTaq DNA polymerase (0.25 µL); template DNA (0.2 µmol/L) and the rest being de-ionized water. The PCR conditions were: 95 °C for 10 min; 35 cycles at 95 °C for 30 s, 57 °C for 30 s, 72 °C for 60 s; and the final extension at 72 °C for 7 min.

The second round of PCR was performed with the 338F-GC, [5′-CGC CCG CCG CGC GCG GCG GGC GGG GCG GGG GCA CGG GGG GCC TAC GGG AGG CAG CAG-3′] and 518r [5′-ATTACCGCGGCTGCTGG-3′] (Muyzer et al. 1993; Liu et al. 2012); this amplified a partial 16S rRNA gene of all the bacteria. The PCR mixture was as mentioned in the first round and the conditions altered only the annealing temperature to 55 °C.

PCR was performed using modified universal primers at the GC clamp of the 5′ terminus for 338F-GC, and 518r to amplify the V3 hypervariable region of the 16S rRNA gene (Muyzer et al. 1993; Liu et al. 2012). PCR products were examined by agarose gel electrophoresis (2% agarose, 1 × TAE) with ethidium bromide staining to confirm product size and viewed under ultraviolet light.

### Bacterial community determination (DGGE)

DGGE (Denaturing gradient gel electrophoresis), of the PCR products obtained in reactions with 338F-GC and 518R primers was performed with slight modification of the adhesive gradient formula by Nanjing New campus patent protocol, (2008), in a polyacrylamide gel (8% w/v) with denaturing gradients containing 50–60% gradient of denaturants (100 mL of 100% denaturants contained 42 g urea, 42 mL of de-ionized formamide, 25 mL of 40% acrylamide/bis [37.5:1] gel monomer, 1 mL of 50 × TAE buffer); using the D-code Universal Mutation Detection System (Bio-Rad Nanjing New Campus Biotechnology Institute, China). Electrophoresis was performed in 1 × TAE buffer at A: 62 V for 1 h and B: 100 V for 16 h in a constant temperature water bath at 60 °C. After electrophoresis, the gel was stained with ethidium bromide (EB) for 30 min followed by distaining with Milli-Q water for 40 min, and the gel was screened with a UV trans-illuminator (Tocan, UVG20; Lunan Wealth Elec MacH IND Co, LTD) to acquire the DGGE image photographed by the Huawei picture app.

To identify the AOB most abundant in the tank systems, we obtained the dominant and best separated DGGE fingerprint bands, cut them out of the gel, extracted the 16S rRNA product using the Sangon Biotech kit following the manufacturer’s instructions, and shipped the product to Sangon Biotech (Shanghai) Co., Ltd; for cloning, sequencing and identification.

### Phylogenetic analysis

The nucleotide sequences for the 16S rRNA genes of the AOB-related bacteria were aligned using CLUSTAL W (Thompson et al. 1994) and compared with available sequences from other members of the ammonia oxidizers in the GenBank database obtained using the Basic Local Alignment Search Tool (BLAST) (Altschul et al. 1997). The nucleotide sequences of the partial 16S rRNA genes from clones of the environmental samples in this study were submitted to GenBank databases and given accession numbers. Phylogenetic reconstructions and evolutionary analyses were conducted in MEGA 7 (Kumar et al. 2016); using the neighbor-joining method (Saitou and Nei 1987) with 1000 bootstrap replicates. The evolutionary distances were computed using the maximum composite likelihood model method (Tamura et al. 2004) and are in the units of the number of base substitutions per site.

### Real-time quantitative PCR

Quantitative PCR of the methane generating AOB 16S rRNA gene was conducted in triplicate using the SYBR Green Real-Time PCR Kit [Bio Rad]. The real-time PCR (qPCR) assays were performed in a CFX96 Touch Deep Well Real-Time PCR Detection system. Primers used in this study were the same as those used for the DGGE of the AOB 16S rRNA gene.

### Data and statistical analysis

One-way ANOVA and Duncan’s multiple range tests, Tukey’s HSD (honestly significant difference), were used to determine mean ± SD; and to test significance of differences amongst the microbial community and physiochemical parameters of water, respectively. The relationships between ammonia-oxidizing bacteria and the physiochemical properties were performed using CANOCO for Windows 4.5. All the variables were normalized via log_10_ (N + 1) transformation, and Monte Carlo permutation tests were used to assess the statistical significance of the relationships.

## Results

### Water quality parameters (environmental characterization)

**Fig. 1:**
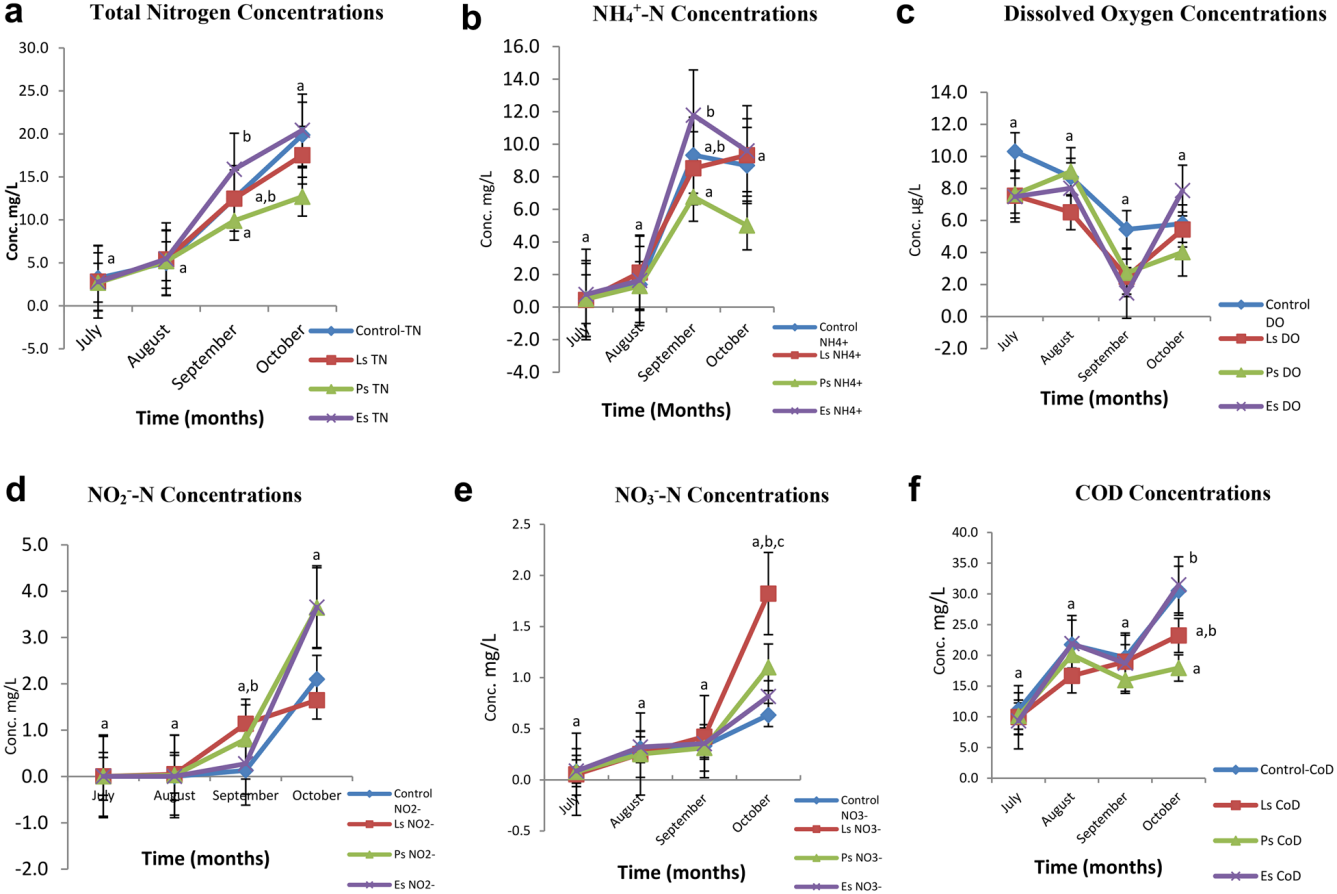
Time-series for water quality parameters observed in the tanks subject to manipulation of microbial communities. Treatments include: the control (*Ctrl*), *Lactic acid* bacteria (*LsB*); *Pseudomonas* (*PsB*) and *Environmental* (*EsB*) bacterial strains. **a**–**f** Present the mean values and standard deviations of 3 replicates (p < 0.05) for TN, NH_4_^+^-N, DO, NO_2_^−^-N, NO_3_^−^-N, and COD concentrations respectively for samples obtained tested the Duncan multiple regression analysis tests in the months of July, August–September and October 2017

The water quality within the twelve tanks was quantified based on parameters TN, NH_4_^+^-N, DO, pH, NO_2_^−^-N, NO_3_^−^N and COD (Additional file 1: Table S1). Concentrations of TN, COD, DO and pH were higher in *EsB* tanks than in the Ctrl tanks; this was followed by *LsB* and *PsB* tanks. Only the NO_3_^−^-N concentration levels were lower (p < 0.05) in *EsB* tanks than in other tanks. This outcome might be attributed to a limited number of heterotrophic microbes breaking down nutrients in the *EsB* tanks compared to the *LsB* and *PsB tanks* which may have contained segmented microbial communities. The TN levels observed for both *LsB* and *PsB* tanks were significantly lower than the Ctrl and *EsB* tanks throughout the experiment. The trend indicates a significant reduction of TN in the order of *PsB*, *LsB*, Ctrl and *EsB* tanks. Our results showed significantly higher levels of TN in *EsB* tanks, while *PsB* tanks had the lowest TN concentration levels in the last 2 months of the experiment (Fig. 1a), which might be attributed to better water quality in these tanks than the other tanks, including limited nitrogenous content. The removal rates of NH_4_^+^-N for the different treatments varied through the different sampling months across all tanks, a significantly high NH_4_^+^-N concentration was observed in the month of September before a drop-off in the month of October. This could be attributed to the temperature exceeding 40 °C in the month of September. Despite temperature changes, *PsB* tank samples had the lowest concentrations of NH_4_^+^-N (p < 0.05). However, *EsB* tanks with the wild microbes and bio-filters revealed contrasting results compared to the Ctrl tanks in (Fig. 1b). The initial DO concentrations in all tanks were considered normal and above the aquaculture standards. As indicated in Fig. 1c, as the study progressed, only the Ctrl tanks DO concentrations were above 5 µg/L throughout the study. More so, in the month of September, the DO concentration in all the remaining tanks was significantly (p < 0.05) affected; for instance *EsB* tanks showed the lowest DO concentrations despite having the highest concentrations in the following months (Fig. 1c). This can be attributed to bacterial activities in the experimental tanks as revealed below.

Furthermore, the levels of NO_2_^−^-N were highest in *LsB* tanks during September when the temperatures were highest. However, in the preceding month (Fig. 1d), the same tank exhibited the lowest concentration (p < 0.05); a pattern attributed to the significant role played by chemotropic microbes breaking down nitrates to nitrites. Interestingly, the nitrate breakdown in the control tanks was higher (p < 0.05) than that in the *PsB* and *EsB* tanks (Fig. 1e). The COD concentrations in the *PsB*, and *LsB* tanks were significantly lower (p < 0.05) than the Ctrl tanks throughout the entire study. However, results from the *EsB* tanks revealed that although the microbial communities breaking down the COD were more active in the initial stages of the culture, attributable to the active growth period, there was less activity in breaking down of the carbon compounds as was observed towards the end (Fig. 1f).

### PCR – DGGE analysis

The first round of amplification using the CTO primers enabled PCR amplification of the 16S rRNA gene in microbes belonging to the ammonia-oxidizing Beta *Proteobacteria* (Kowalchuk et al. 1997; Ziembinska et al. 2009); and the resultant product was re-amplified to generate the second (next) generation product. Furthermore, a second round of amplification using the 338F-GC and 518r primers was performed to amplify the partial 16S rRNA gene of all the bacteria (Muyzer et al. 1993), after which the bands were observed under UV light. The fingerprint of the obtained sample product (fig not shown), the most clear bands, were cut out for DGGE analysis.

**Fig. 2:**
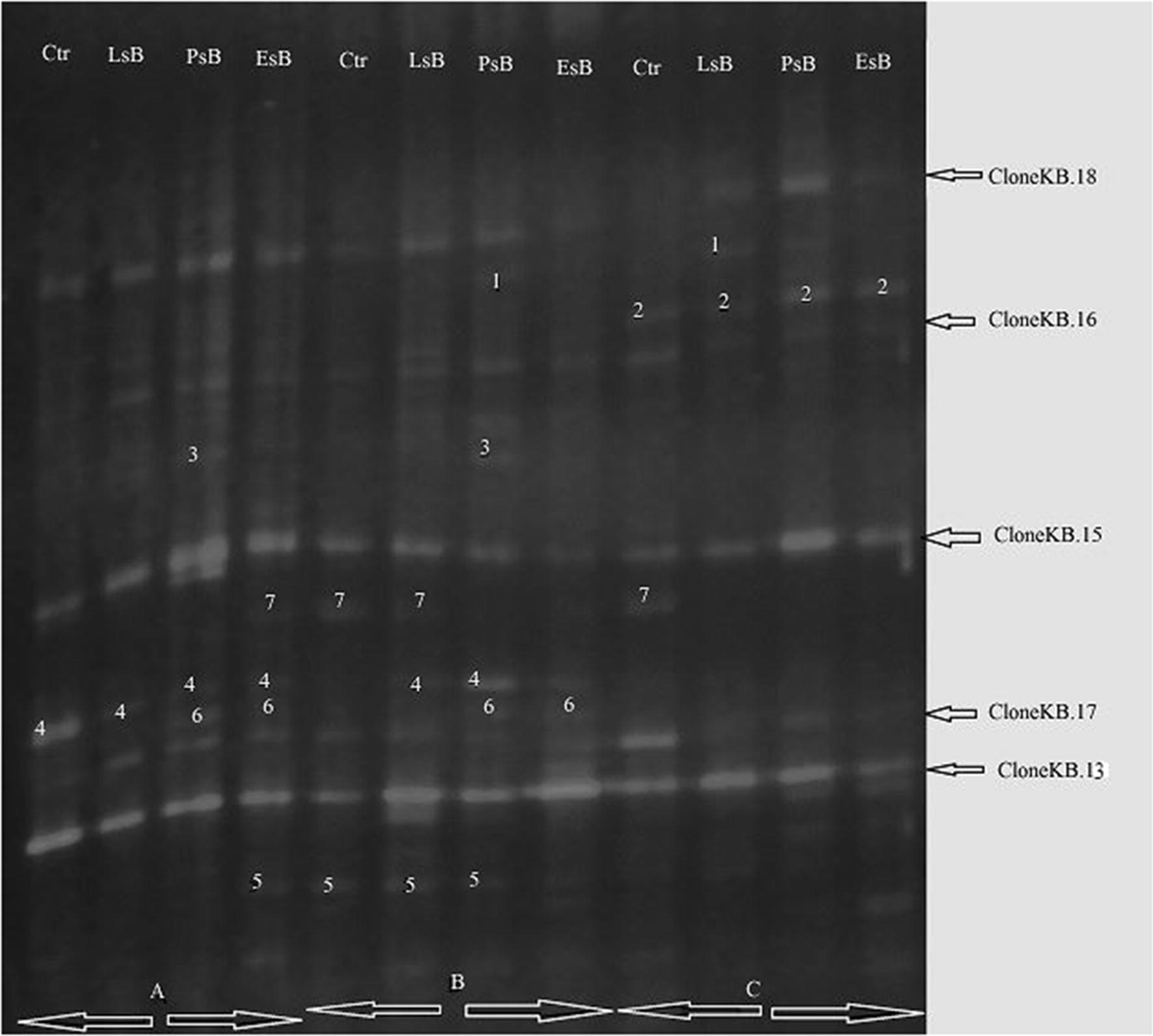
DGGE profiles showing prominent amplicons from AOB communities in tanks *Ctrl; LsB; PsB* and *EsB*. Clones; KB13, KB.15 to KB.18 and numbers 1–7 are the observed and sequenced bands between 170 and 200 bps for 16S rRNA gene. *Ctr, Ls, Ps,* and *Es* denote control (*Ctr*)*; Lactic acid bacterial* strain (*LsB*)*; Pseudomonas bacterial* strain *(PsB)* and *Environmental bacterial* strain (*EsB*). A, B, and C represent the sampling months of August, September and October, respectively

To investigate changes in microbial community structure during the enrichment process, DGGE analysis of second generation PCR product was performed, and the results are presented in Fig. 2. Clear differences between the microbial community structures within the different treatment tanks in space and time were observed. Figure 2 shows the different bands for the ammonia-oxidizing bacteria obtained after DGGE of all three sampling times performed. Our results of the AOB fingerprint bands revealed that the most prominent bands lay within a similar range of 170–200 bps. The average number of recognized DGGE bands from the four treatment tanks throughout the study period were; 10, 10, 11 and 12 bands for the Ctrl, *LsB*, *PsB* and *EsB* tanks respectively. The sequences obtained were identified by comparison with sequences in the NCBI database using BLAST and then forwarded to Gen-Bank for (Accession Numbers MG807409-MG807414).

**Fig. 3:**
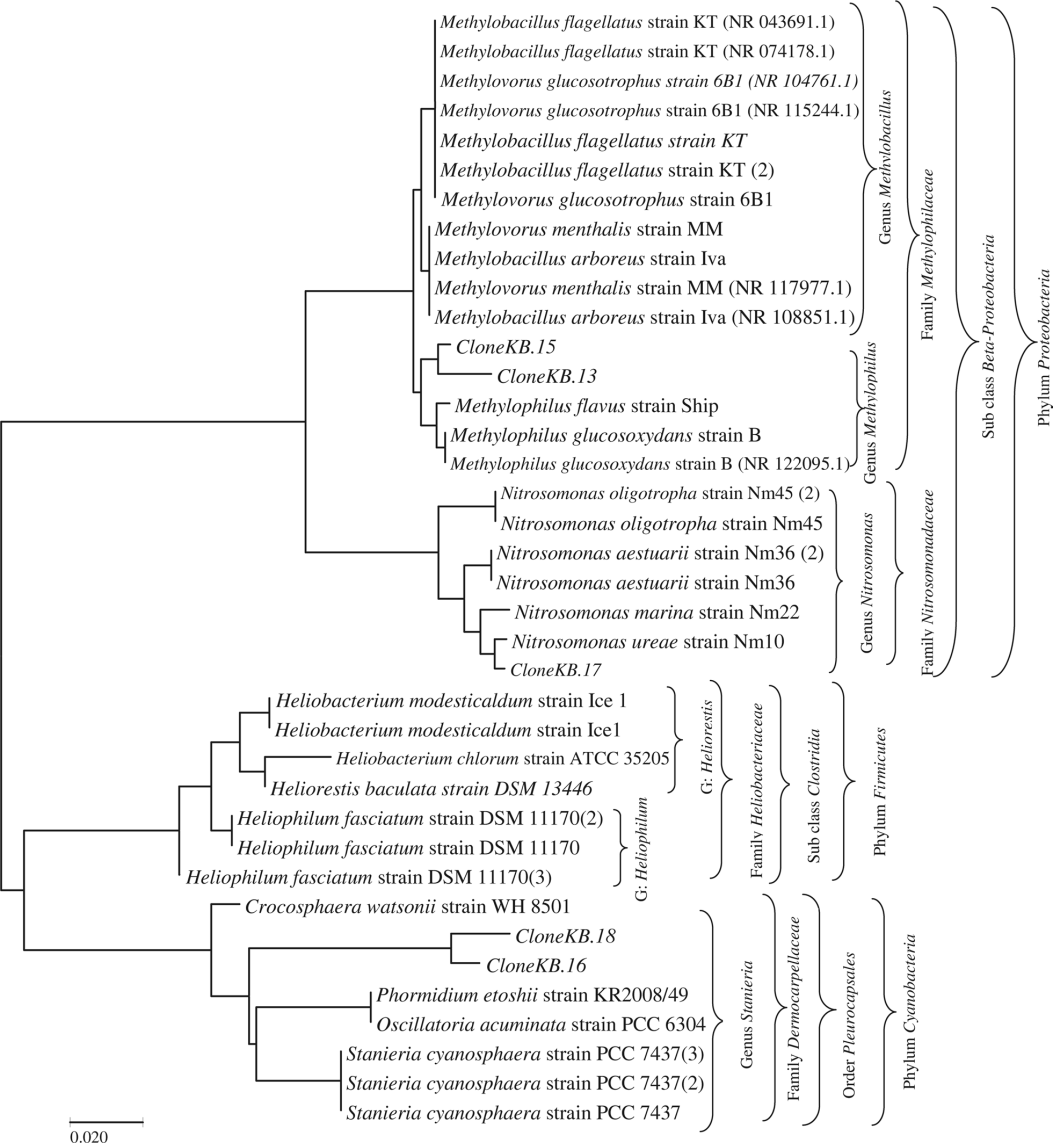
Evolutionary relationships among taxa for the dominant AOB communities based on the nearly full-length 16S rRNA gene sequences specific for AOB. The five dominant microbial genes sequences i.e., Clones; KB.13, KB.15, KB.16, KB.17 and KB.18 are classified to their most probable genera inferred using the Neighbor-Joining method, conducted in MEGA7

DGGE profiles of the AOB community structures in the tanks revealed a pattern of AOB 16S rRNA products 5 dominant bands that were similar and clearly observed in all the tanks, with consistent appearance at the different sampling times. As shown in Fig. 3, they were labeled cloneKB.13 (MG807410), cloneKB.15 (MG807411), cloneKB.16 (MG807412), cloneKB.17 (MG807413) and cloneKB.18 (MG807414). Upon phylogenetic analysis, the sequences revealed affiliations with environmental samples of genera *Methylobacillus, Stanieria, Nitrosomonas, and Heliorestis*. Furthermore, two other bands were identified at specific times and in specific tanks. For instance, band 7 (cloneKB.14), seen to be dominant with intensification during the enrichment and as the study progressed was identified as *Micrococcus aloeverae strain AE*-*6.* Band 2, which emerged during later stages of the enrichment process, was identified as *Kinneretia asaccharophila*. Both conditions could be attributed to adaptation and effective utilization of nitrates under aerobic conditions during selective enrichment (Hilyard et al. 2008; Chanika et al. 2011; Yao et al. 2013).

**Fig. 4:**
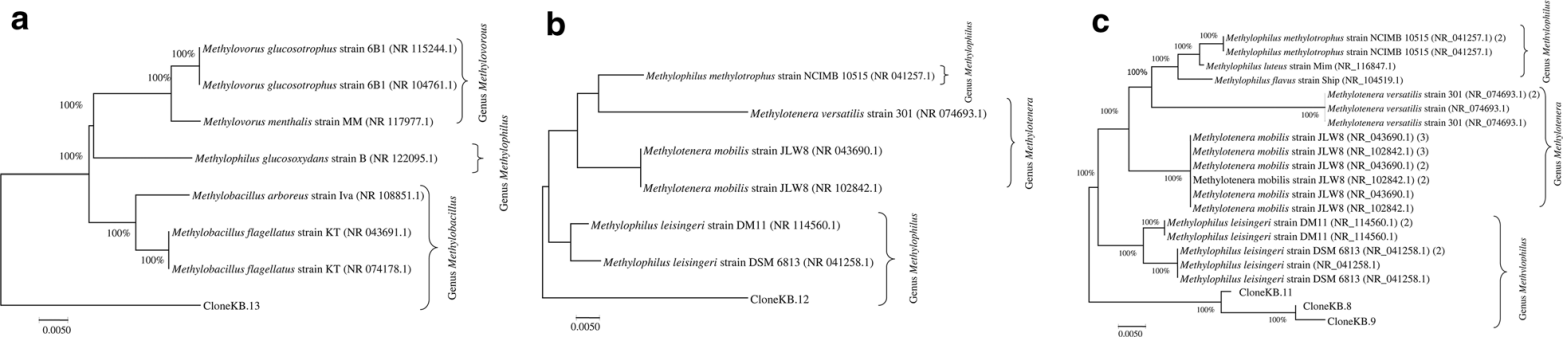
Phylogenetic trees for the unique AOB communities in the enriched tanks *LsB* and *PsB* based on the nearly full-length 16S rRNA gene sequences. **a**, **b** Microbial communities sampled in tanks *LsB* and *PsB* respectively. **c** The strains that were observed at specific time under the different treatments

Of the 10 different bands sequenced, six were identified to their closest phylogenetic affiliation from the *Proteobacteria* phylum and sub-class *Beta*-*Proteobacteria*, while the others were from the phyla *Actinobacteria*, *Cyanobacteria* (2) and *Firmicutes*, which were further identified at species levels (Table 1). Basing on the identification of microbial genes from *LsB*, *PsB* and *EsB* tanks with a phylogenetic analysis revealing specific AO nitrifiers, we suspect that there are methanogenic ammonia oxidizing bacteria present, and exhibited in different compositions and distribution patterns (Supplementary data SD1). The *LsB* tank enriched with LAB revealed two dominant strains phylogenetically identified as *Methylobacillus arboreus strain Iva* and *Methylobacillus flagellates strain KT* (Fig. 4a), while the clone KB.12 from the *PsB* tank enriched with the *Pseudomonas* strain was closely related to the *Methylophilus methylotrophus strain NCIMB 10515 species* (Fig. 4b).

**Table 1:** Classification and taxonomy of identified bacterial microbes of 16S rRNA gene obtained from treatment tanks *Ctrl; LsB; PsB* and *EsB* operating under bio-filter technologies

Domain	Class	Order	Family	Genus	Species	NCBI Accession
Bacteria	*Betaproteobacteria*	*Burkholderiales*	*Comamonadaceae*	*Kinneretia*	*Kinneretia asaccharophila* sp.	MG807399
Bacteria	*Betaproteobacteria*	*Nitrosomonadales*	*Methylophilaceae*	*Methylotenera*	*Methylotenera mobilis* sp.	MG807408
Bacteria	*Betaproteobacteria*	*Nitrosomonadales*	*Methylophilaceae*	*Methylophilus*	*Methylophilus leisingeri* sp.	MG807409
Bacteria	*Betaproteobacteria*	*Nitrosomonadales*	*Methylophilaceae*	*Methylobacillus*	*Methylobacillus arboreus* sp.	MG807410
Bacteria	*Betaproteobacteria*	*Nitrosomonadales*	*Methylophilaceae*	*Methylobacillus*	*Methylobacillus arboreus* sp.	MG807411
Bacteria	*Betaproteobacteria*	*Nitrosomonadales*	*Nitrosomonadaceae*	*Nitrosomonas*	*Nitrosomonas oligotropha* sp.	MG807413
Bacteria	*Actinobacteria*	*Micrococcales*	*Micrococcaceae*	*Micrococcus*	*Micrococcus aloeverae* sp.	NR_134088.1
Bacteria	*Cyanobacteria*	*Pleurocapsales*	*Dermocarpellaceae*	*Stanieria*	*Stanieria cyanosphaera* sp.	MG807412
Bacteria	*Cyanobacteria*	*Chroococcidiopsidales*	*Chroococcidiopsidaceae*	*Chroococcidiopsis*	*Chroococcidiopsis thermalis* sp.	NR_102464.1
Bacteria	*Clostridia*	*Clostridiales*	*Heliobacteriaceae*	*Heliorestis*	*Heliorestis baculata* sp.	MG807414

The cloning, sequencing and identification were done at to Sangon Biotech (Shanghai) Co., Ltd; and sequences deposited at NCBI for obtaining accession numbers

Fingerprint bands that were clear in some samples—such as bands 3, 4, and 5 (Fig. 2)—which identified closest to *Methylophilus leisingeri*, *Methylotenera mobilis* and *Methylophilus methylotrophus* strains, respectively (Fig. 4c), became less intense or disappeared in the profile of subsequent sampling in the same tanks. The observed patterns and distributions could well be associated with the biodegradation of organic content or the effect of the bio-filter colonization in the tanks.

Interestingly, two other clear bands—labeled 6 and 7—were identified as shown in Fig. 2. This enriched our study in revealing significant differences in the ammonia oxidizing bacterial strains from the different tanks; however, after sequencing, the microbes identified as cloneKB.5 and cloneKB.14 identified closest to *Chroococcidiopsis thermalis* sp. and *Micrococcus aloeverae* strain of classes *Cyanobacteria* and *Actinobacteria* respectively. This result confirmed that CTO primers may not only amplify 16S rRNA of ammonia-oxidizing bacteria, but of any bacteria possessing similar sequences.

### Real-time quantitative PCR

**Fig. 5:**
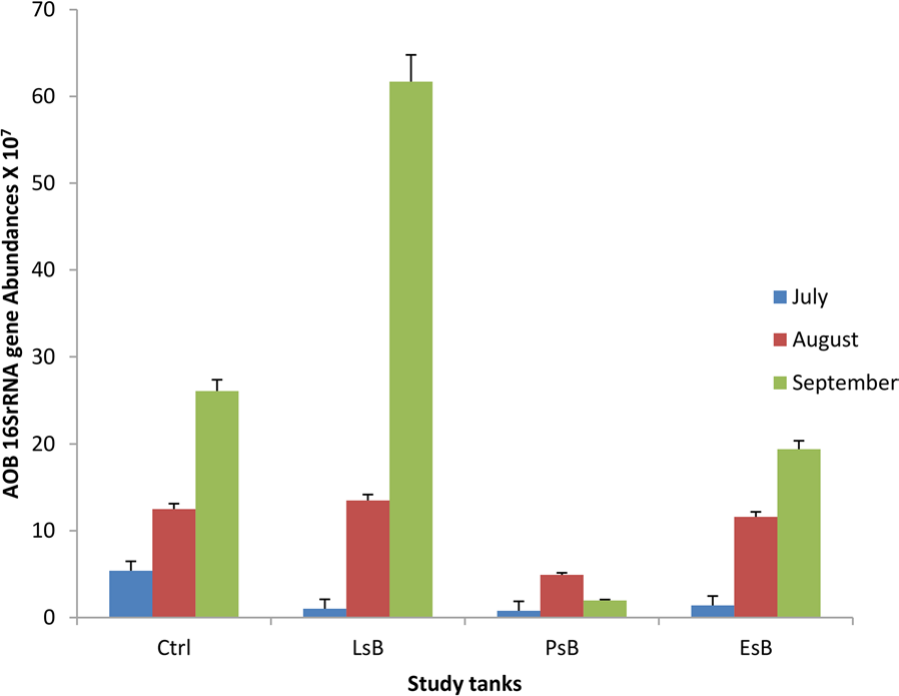
The methane-generating ammonia-oxidizing 16S rRNA gene abundances in the tanks for production systems fitted with bio-filters. The figure represents the average CFU values and standard deviations of 3 replicates (p < 0.05) for *Ctrl, LsB, PsB,* and *EsB* respectively, showing abundance values for samples obtained after real-time qPCR analysis tests in the months of July, August and September 2017

Results from the real-time qPCR of the 16S rRNA gene revealed that the ammonia-oxidizing microbial gene expression and abundances observed in this study after acclimatization and experimental run of our samples showed a higher bacterial abundance in the control tanks than the other tanks (Fig. 5). Tank *EsB*, which had no enrichments but was fitted with bio-filters, had lower microbial abundances too, suggesting a significant influence of the bio-filters on the bacterial communities. Furthermore, the results revealed a trend of three treatments’ tanks being similar (i.e. tanks *Ctrl, LsB* and *EsB*), while the last tank, (*PsB*), was contrary to the other results. For the former 3 tanks, the bacterial content increased with time, showing a very high significant difference (p < 0.05) in distribution in the tank *LsB* in September during our sample analysis. Meanwhile in the latter tank, *PsB*, the microbial abundances increased in August and then significantly decreased (p < 0.05) in the last month of sampling. These abundances did correlate to the water quality parameters suggesting that other factors, like temperature, or bio-filter colonization were the most likely factors affecting the *Pseudomonas* strain microbial abundances and survival.

### Relationship between AOB community composition and environmental quality

**Fig. 6:**
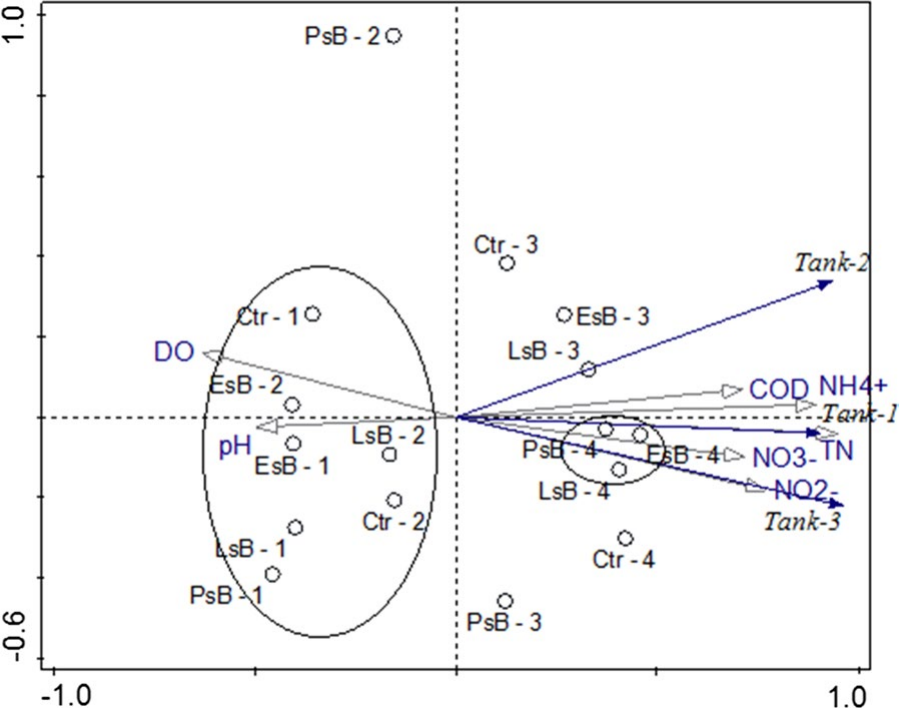
Principal coordinate analysis (PCA) ordination diagrams for AOB communities associated with the water quality parameters. The figure presents species and water quality correlations. Water quality parameters are indicated as arrows and the microbial communities indicated as (o). The first canonical axes for the microbial communities explained 70.5, 96.4, 98.8 and 99.7% of the variations for the: control (*Ctrl, LsB, PsB* and *EsB*) treatment tanks respectively

To determine the effects of AOB community composition on water quality, the relationship among the four treatments, microbial communities and environmental parameters was analyzed using principle component analysis (PCA) ordination, as shown in Fig. 6. Identifying and defining the environmental characteristics that drive the bio-filter technologies colonized by microbial communities used in denitrifying aquaculture systems. Total variables analyzed accounted for 90.9% variations with explained variation of 80.5% after adjustments. Determining the effective extent of environmental characteristics on AOB microbial community by PCA revealed significant relationships with the first canonical axes for the AOB fingerprints explaining 70.5, 96.4, 98.8 and 99.7% of the cumulative variances of the species data in tanks Ctrl, *LsB*, *PsB* and *EsB* respectively, while the Eigen values summed up to 1.0 and corresponded with 0.817, 0.148, 0.021 and 0.012 for the respective tanks. From our results, Fig. 6, showed that all results obtained after the first and second sampling of all tanks e.g. *Ctr*-*1, LsB*-*1, Ctr*-*2,* and *PsB*-*2* in the months of July and August were projected in the opposite direction of the steepness for the environmental parameters with the exception of the physiological parameters (i.e., DO and pH). All treatment tanks, in the third (*Ctr*-*3, LsB*-*3, PsB*-*3* and *EsB*-*3*) and fourth (*Ctr*-*4, LsB*-*4, PsB*-*4* and *EsB*-*4*) month after sampling the water quality parameters i.e., NO_2_^−^, NO_3_^−^, NH_4_^+^, TN and COD, exhibited a more positive correlation to the microbial communities than did for pH and DO, even though in tanks *PsB*-*4* and *EsB*-*4* it was observed that TN and NO_3_^−^ may have had a more influencing microbial-water quality relationship. The order of correlation with the water quality parameters was NO_3_^−^, NO_2_^−^, TN, NH_4_^+^ and COD respectively.

## Discussion

The ammonia oxidizing nitrifiers in marine and freshwater aquaculture systems have been studied and a great wealth of knowledge is documented e.g. Huang et al. (2018) in their study on: “Ammonia oxidizing bacteria and archaea within bio-filters of a commercial re-circulating marine aquaculture system; Brown et al. (2013) study on ammonia-oxidizing archaea and nitrite-oxidizing *nitrospira* sp. in the bio-filter of a shrimp re-circulating aquaculture system; Coci et al. (2004) study on denaturing gradient gel electrophoretic analysis of ammonia-oxidizing bacterial community structure in the lower Seine river: Impact of Paris wastewater effluents; and Sauder et al. (2011) study on Aquarium nitrification revisited: *Thaumarchaeota* are the dominant ammonia oxidizers in freshwater aquarium bio-filters” etc. However, the mechanism and mode of operation of their studies in relation to the bio-filter technology is not clearly understood. To get a vivid understanding of such technologies, our study focused on characterizing some AO microbes for biological water waste cleaning in aquaculture production systems. In this study, experimental results identified the AO nitrifiers with the intention of getting an in-depth knowledge on the effect and relationships of bio-filter technology on a group of microbial communities that have a methyl group in the partially anaerobic ammonia oxidation pathway. Our results revealed the bio-filter microbial communities, operating mechanisms and correlations in both bio-filter and enrichment technologies as used in the same production system. Keppler et al. (2006) argue that methane production occurs in oxygenated environments under ambient conditions, although they also suggest that this process may involve non-microbial methane generation from plant matter, with temperature and ultraviolet light as key factors affecting this process. Furthermore, Karl et al. (2008) and Metcalf et al. (2012) suggest that methane production under aerobic conditions is possible in a process involving degradation of methylphosphonate in near-surface ocean water. This could explain the identified methyl ammonia oxidizer communities found in the fish tanks that are not anoxic, but oxygenated to keep the fish alive.

From our results, microbial communities significantly influenced the water quality parameters in both predictable and unpredictable trends as revealed in the differences (p < 0.05) in the end products in the operation of the two different technologies (i.e., bio-filter and microbial enrichment). The control tanks containing wild microbial communities, with no enrichment microbes or suspended bio-filters were expected to have the highest concentrations of various nutrients during the environmental measurements. From our findings, there were differences observed in the tank setups, suggesting the influence of both technologies to improvement of water quality. The *EsB* tanks, containing wild microbial communities and fixed bio-filters had the highest levels of most measured water quality parameters except for NO_3_^−^-N. This finding implies that the rate of nutrient breakdown in these tanks was significantly lower than the treatments including the control even in the presence of bio-filters. In relation to Carlson and Amy (1998), findings in which biological organic material (BOM) was removed during bio-filtration, they observed that organic removal in a bio-filter is limited either by biodegradable organic matter formation or biomass concentration and not by filter operating parameters (Bouwer and Crowe 1988). Therefore, in our findings, we attributed these results to the levels of the heterotrophic bacteria that may have been compromised in the initial stages, and may not have taken up sites for colonization and regeneration. Furthermore, two types of aerobic microorganisms colonize bio-filters for aquaculture, heterotrophic bacteria (e.g., *Nitrospira* sp.) which utilize the dissolved carbonaceous materials as a food source to form nitrates, and chemotrophic bacteria (e.g., *Nitrosomonas* sp.) that utilize ammonia as a food source to produce nitrite as a waste product. Chemotrophic microbes grow and colonize the bio-filter as long as there is a food source, but they are relatively slow growers; whereas heterotrophic microbes grow five times faster and out-compete others for space (Chaudhary et al. 2003). Also, because the filters are suspended in the tank, and there is limited control of the carbonaceous, BOD before water passes through the filters, heterotrophs settle on the substrates much faster.

In related studies, temperature is reported to have no significant effect on the composition of the microbial community if the temperature changes are minimal for 4 weeks or less (Avrahami et al. 2003). However, over longer periods or if temperatures drastically rise, like in our study; temperatures might indirectly affect the communities. In the months of September, the temperature rose to 40 °C, which may have indirectly affected the microbial communities that directly affected DO, TN, and NH_4_^+^-N content especially in the *EsB* tank reducing (p < 0.05) removal rates of the respective water quality parameters. The levels of DO in September were significantly reduced, an observation attributed to biodegradation, thus we infer higher utilization of dissolved oxygen across all tanks in relation to the microbial densities. This could also be due to the experimental fish increasing utilization of dissolved oxygen. On the contrary, the COD concentrations in the month of September reduced significantly within all tanks, but had a higher reduction in tank *PsB*.

Basing on the results from the real-time qPCR, (Fig. 5) of the 16S rRNA gene of the AO microbial abundances, the enrichment and bio-filter colonization technologies revealed positive trends in bioaccumulation, just as other studies suggest, (Huang et al. 2018; Wang et al. 1995; Ahmad and Amirtharajah 1998; Carlson and Amy 1998). However, tank *PsB* containing the *Pseudomonas* strain did reveal a significant effect on the microbial abundances. Temperature peaks to over 40 °C probably had the most significant factor causing the reduction of the microbial counts.

From the results of Fig. 6, the arrow heads revealed the direction of the steepest increment in the water quality variables for the former and the corresponding treatments for the latter. The length of the arrow is a measure of fit for the variables i.e. water quality or treatments, and it also predicts the multiple correlations of the variable with the ordination axes. Furthermore, we find that the projection points lying on opposite directions predict negative correlations, which suggests that parameters studied at that time did not significantly affect the microbial community. This assumption is factored in as we related the finding to the conclusions of Carlson and Amy (1998), that in the first 2 months, bacteria are growing and forming microbial mass to attach onto the filter media as bio-film, oxidizing most of the organics and using them as an energy supply and carbon source. This may not be the same within our four treatment ecosystems as the entire biological communities interact in the following months. This was in agreement with studies by Ahmad and Amirtharajah (1998) and Servais et al. (1994) who revealed that within natural surface water, a period of 3 months is required for a granular activated carbon (GAC) filter to retain maximum amounts of biomass. Similarly, the correlation was determined depending on the sharper angles less than 90° suggesting a positive correlation, while the result of a projection line ending at the ordination origin (zero point) correlation is predicted to be zero.

### Bacterial communities

In this study, a nested-PCR design focused on the amplification of the 16S rRNA gene in the ammonia-oxidizing bacteria involving a first round of PCR with CTO primers then a second round with 338F-GC and 518r primers, allowing increment in the sensitivity of amplification for the specific V3 DNA region (Muyzer et al. 1993; Ward et al. 1997; Boon et al. 2001; Dar et al. 2005; Ziembinska et al. 2009). We also used DGGE, a known useful method for monitoring bacterial communities (Muyzer et al. 1993; Konneke et al. 2005) enabling extraction of the dominant DNA bands from the fingerprint for sequencing and identification of the most abundant bacteria in the different communities.

From our findings, the majority of microbes identified are from phylum *Proteobacteria* that are widely established in freshwater environments and play important roles in the process of nutrient cycling and mineralization of organic compounds (Kersters et al. 2006; Cardona et al. 2016; Deng et al. 2018). The dominant genera of ammonia-oxidizing nitrifiers have been extensively studied and are responsible for efficient nitrification, such as *Nitrosomonas* sp. (Ziembinska et al. 2009), *Nitrosomonas europea* (Juretschko et al. 1998; Wagner et al. 2002), and *Nitrospira* sp. (Itoi et al. 2007). This study discussed the profiles of the other AOB communities generated using DGGE to assess the expression of 16S rRNA genes followed by DNA sequencing that may differ from the profiles above. Two different bacterial enrichments, i.e. tank *LsB* containing LAB, and tank *PsB* containing a *Pseudomonas* strain, alongside a tank *EsB* containing wild microbial type are fitted with fixed-film submerged aerobic bio-filters against a control tank containing only wild microbes. The twelve tanks were used to investigate the capability of AOB microbes in summer production in tilapia tanks. From molecular study literature, ammonia-oxidizing bacteria genera are classified under the Beta- and Gamma-*Proteobacteria*, thus limiting our understanding to these two phylogenetic groups (Egli et al. 2001; Konneke et al. 2005). Our discussion is on the characterization, identification and isolation of the methyl ammonia-oxidation bacteria considered in a newly discovered pathway that combine the generation of methane and the microbial nitrogen cycle, allowing ammonia to be oxidized to nitrite or nitrate under aerobic and anoxic conditions. We found evidence that the distribution of methane and nitrification activities in intensive production systems relate to similarities between nitrifiers and methanotrophs. Therefore, characterizing the ammonia oxidizing nitrifiers involved in the intensive tank and pond production would support our suggestion that the role of conventional methanotrophs is partially fulfilled by other kinds of bacteria, ammonia-oxidizing nitrifiers involved in the methane metabolism.

Figure 2, obtained from DGGE analysis after the second round of PCR, showed substantial differences in the microbial communities. Changes in the microbial community structures for the different tanks in both space and time were observed. The environmental samples identified as clones KB.13, KB.15, KB.16, KB.17 and KB.18 showed resilience and dominance in appearance throughout the entire study. Results of the phylogenetic analysis based on 16S rRNA gene sequences show that the clones respectively belonged to *Methylobacillus* sp.*, Methylobacillus* sp.*, Stanieria* sp.*, Nitrosomonas* sp. and *Heliorestis* sp. We further identified the sequences of the other microbes with clear fingerprints despite appearing at specific times an effect we attributed to environmental characteristics such as bio-filtration, succession and temperature variations. Band 4, named cloneKB.9, identified as *methylophillus mobilis* was found only in the initial stages and disappeared in the last month of sampling. This could be due to un-conducive environmental characteristics for the communities’ survival. Bands 3 and 5 identified as *Methylophilus leisingeri* and *Methylophilus methylotrophus* also were found at one stage of the culture and not throughout, which could be attributed to failure of attachment on the bio-filters for colonization. Meanwhile band 2, named cloneKB.1 and identified as *Kinneretia asaccharophila* sp., was observed in all tanks at later stages of the study after subsiding of the extreme temperatures; this was attributed to conducive conditions for succession of microbes to emerge and thrive in the later stages.

Bacterial bands 6 and 7, denoted as clones KB.5 and KB.14, were identified to be closest to *Chroococcidiopsis thermalis strain PCC 7203* (NR_102464.1) and *Micrococcus aloeverae strain AE*-*6* (NR_134088.1). These may not specifically be ammonia-oxidizing nitrifiers but owning to the fact that the tanks were outdoors, it’s highly possible that these species from the *cyanobacterial* and *Actinobacteria* sub-classes drifted into the system. Their identification would be caused by the non-specificity of the CTO primer sequences, leading to an amplification of the 16S rRNA genes possessing similar primer binding sequences belonging to the beta-*Proteobacteria* sub-class that may not necessarily be ammonia-oxidizing nitrifiers. Other researchers—like Purkhold et al. (2000), Ziembinska et al. (2009), and Song et al. (2013)—also suggested that in such situations, one could confirm that there are no known primers that can amplify only the AOB 16S rRNA gene. Coci et al. (2004) study on: “denaturing gradient gel electrophoretic analysis of ammonia-oxidizing bacterial community structure in the lower Seine River: Impact of Paris wastewater effluents”, did prove there was non-specificity of the CTO primers since none of the published primers intended to target all β-subclass ammonia oxidizing bacteria showed 100% sensitivity or specificity.

From the entire study, we observe that the abundant AO nitrifiers belong to the beta and Gamma sub-classes of the *Proteobacteria*, and some oxidizers contained a methyl group that is known to be representative of a new pathway for electron donation for the formation of methane gas. The identified AO nitrifiers using substrates with the methyl group did play the role of conventional methanotrophs, resulting in methane metabolism, confirming that nitrifiers are involved in intensive tank and pond production, and the methane cycle in freshwater as well.

The performance of a bio-filter will depend greatly on the biomass attached to the filter media and biomass growth. Its maintenance over the surface of the filter media, on the other hand, may depend on the surface characteristics of the filter medium itself, which highly affects biomass growth rate and biomass retention capacity. This may explain the deviation from expected sizes for the bacterial compositions in our final findings, which revealed anomalies in microbial contents in the different tanks even though a standard specific amount of microbes were enriched at a specific time.

Similarly, the biomass accumulation could have been affected by filtration rates, and filter backwashing mechanisms in each study tank. In this study, focus was on the characterization and identification of microbes thriving under this technology; hence, we call for further investigations.

In conclusion, five dominant bands obtained after DGGE profiling of aquaculture bio-filter microbial communities were characterized and found to closely relate to uncultured environmental samples of genera *Methylobacillus, Stanieria, Nitrosomonas, and Heliorestis*. Some of the identified bacterial communities revealed a methyl ammonia group. Temperature rises to about 40 °C significantly affected the environmental characteristics, especially DO, TN and NH_4_^+^-N that might have directly or indirectly affect the microbial communities. Finally, a nested PCR design was preferred for its ability to allow increased sensitivity of amplification for the specific DNA region even in limited amounts. We observed that CTO primers may not amplify only 16S rRNA of ammonia-oxidizing bacteria, but do any bacteria possessing similar sequences. Therefore, there is need to carry out further studies to obtain a more precise answer to this question.

## Additional file

**Additional file 1: Table S1.** Water quality parameters for tanks containing the control (*Ctrl); Lactic acid bacterial* strain *(LsB); Pseudomonas* bacterial strain *(PsB) and Environmental bacterial* strain *(EsB).* Data are expressed in mean ± standard deviations (M ± SD) from triplicate tanks (p < 0.05) for samples obtained according to Duncan multiple regression analysis tests and Tukey’s HSD in the months of July, to October 2017. Values with the different superscript letters in the same column and month are significantly different (p < 0.05) from the control (*Ctrl*).

## Abbreviations

Anammox: anaerobic ammonia oxidizing
PCR: polymerase chain reaction
DGGE: denaturing gradient gel electrophoresis
TN: total nitrogen
COD: chemical oxygen demand
BOD: biological oxygen demand
AOB: ammonia oxidizing Bacteria
DO: dissolved oxygen
FCR: feed conversion ratio
NH_4_^+^-N: ammonium nitrogen
NO_2_^−^-N: nitrite nitrogen
NO_3_^−^-N: nitrate nitrogen
(NH_4_)_2_SO_4_: ammonium sulphate
qPCR: real-time quantitative polymerase chain reaction
pH: hydrogen ions
*Ctrl* tank: control tank
*LsB* tank: *lactic acid bacterial* strain tank
*PsB* tank: *Pseudomonas bacterial* strain tank
*EsB* tank: *environmental bacterial* strain tank
LAB: lactic acid bacteria
