# Supplementary material for: Methane-generating ammonia oxidizing nitrifiers within bio-filters in aquaculture tanks
Source: AMB Express. 2018 Aug 28;8:140. doi: 10.1186/s13568-018-0668-2 (PMC6113197; doi:10.1186/s13568-018-0668-2)
Supplement: Supplementary file 1 — Additional file 1: Table S1. Water quality parameters for tanks containing the control (Ctrl); Lactic acid bacterial strain (LsB); Pseudomonas bacterial strain (PsB) and Environmental bacterial strain (EsB). Data are expressed in mean ± standard deviations (M ± SD) from triplicate tanks (p < 0.05) for samples obtained according to Duncan multiple regression analysis tests and Tukey’s HSD in the months of July, to October 2017. Values with the different superscript letters in the same column and month are significantly different (p < 0.05) from the control (Ctrl). [file 13568_2018_668_MOESM1_ESM.docx]

Additional file 1: Table S1

| Month | **Tank Grouping** | **TN**  **(mg/L)**  **Mean ± SD** | **NH_4_^+^**  **(mg/L)**  **Mean ± SD** | **NO_2_^-^**  **(mg/L)**  **Mean ± SD** | **NO_3_^-^**  **(mg/L)**  **Mean ± SD** | **COD**  **(mg/L)**  **Mean ± SD** | **DO**  **(ug/L)**  **Mean ± SD** | **pH**  **Units**  **Mean ± SD** |
| --- | --- | --- | --- | --- | --- | --- | --- | --- |
| July | Control - (Ctrl) | 3.224 ± 0.55 | 0.520 ± 0.23 | 0.003 | 0.082 ± 0.09 | 11.079 ± 3.45 | 10.305 ± 2.35 | 8.945 ± 0.23 |
|  | *Lactic acid bacterial* strain *- (LsB)* | 2.820 ± 0.41 | 0.442 ± 0.42 | 0.003 | 0.055 ± 0.07 | 9.951 ± 2.97 | 7.547 ± 2.61 | 8.382 ± 0.52 |
|  | *Pseudomonas* strain *- (PsB)* | 2.692 ± 0.66 | 0.488 ± 0.40 | 0.004 | 0.078 ± 0.09 | 10.111 ± 4.09 | 7.637 ± 2.44 | 8.395 ± 0.68 |
|  | *Environmental microbes - (EsB)* | 2.778 ± 1.13 | 0.776 ± 0.71 | 0.004 | 0.087 ± 0.08 | 9.337 ± 3.75 | 7.487 ± 2.27 | 8.940 ± 0.21 |
| August | Control *- (Ctrl)* | 5.009 ± 1.83 | 1.389 ± 1.51 | 0.002^a^ | 0.308 ± 0.07 | 21.743 ± 10.68 | 8.709 ± 3.25 | 8.182 ± 0.46^a,b^ |
|  | *Lactic acid bacterial* strain *- (LsB)* | 5.397 ± 2.07 | 2.119 ± 1.66 | 0.050 ±0.073^b^ | 0.253 ± 0.11 | 16.658 ± 3.63 | 6.511 ± 4.61 | 7.762 ± 0.58^a^ |
|  | *Pseudomonas* strain *- (PsB)* | 5.193 ± 2.25 | 1.293 ± 1.27 | 0.028 ± 0.05^a,b^ | 0.252 ± 0.16 | 20.063 ± 5.34 | 9.057 ± 4.53 | 8.227 ± 0.54^b^ |
|  | *Environmental microbes - (EsB)* | 5.458 ± 1.92 | 1.641 ± 1.51 | 0.004^a^ | 0.322 ± 0.18 | 21.913 ± 5.10 | 8.013 ± 4.96 | 8.063 ± 0.60^a,b^ |
| September | Control - *(Ctrl)* | 12.502 ± 3.56^a,b^ | 9.327 ± 2.74^a,b^ | 0.132 ± 0.18^a^ | 0.334 ± 0.14 | 19.639 ± 5.37 | 5.442 ± 6.36 | 7.779 ± 0.78 |
|  | *Lactic acid bacterial* strain *- (LsB)* | 12.468 ± 4.31^a,b^ | 8.526 ± 4.60^a,b^ | 1.139 ± 1.51^b^ | 0.423 ± 0.25 | 18.963 ± 6.77 | 2.488 ± 3.96 | 7.437 ± 0.50 |
|  | *Pseudomonas* strain *- (PsB)* | 9.899 ± 4.26^a^ | 6.766 ± 3.29^a^ | 0.807 ± 0.77^a,b^ | 0.313 ± 0.18 | 15.954 ± 2.54 | 2.732 ± 2.67 | 7.499 ± 0.43 |
|  | *Environmental microbes - (EsB)* | 15.885 ± 3.76^b^ | 11.784 ± 2.96^b^ | 0.275 ± 0.62^a,b^ | 0.355 ± 0.13 | 18.710 ± 3.79 | 1.472 ± 1.23 | 7.411 ± 0.30 |
| October | Control *- (Ctrl)* | 19.877 ± 2.99 | 8.695 ± 4.19 | 2.100 ± 1.84 | 0.635 ± 0.39 | 30.510 ± 2.56^b^ | 5.800 ± 3.66 | 8.265 ± 0.49 |
|  | *Lactic acid bacterial* strain *- (LsB)* | 17.525 ± 12.29 | 9.328 ± 10.30 | 1.648 ± 1.87 | 1.823 ± 2.36 | 23.235 ± 6.70^a,b^ | 5.432 ± 4.06 | 8.122 ± 0.49 |
|  | *Pseudomonas* strain *- (PsB)* | 12.699 ± 6.33 | 5.011 ± 4.12 | 3.645 ± 3.93 | 1.102 ± 0.89 | 17.937 ± 5.86^a^ | 4.017 ± 0.91 | 7.922 ± 0.14 |
|  | *Environmental microbes - (EsB)* | 20.434 ± 8.50 | 9.589 ± 7.00 | 3.656 ± 2.97 | 0.817 ± 0.68 | 31.466 ± 12.26^b^ | 7.867 ± 4.11 | 8.430 ± 0.69 |
|  |  |  |  |  |  |  |  |  |
